# Complete chloroplast genome of *Lonicera crassifolia* Batalin (Caprifoliaceae) and its phylogenetic implications

**DOI:** 10.1080/23802359.2022.2068979

**Published:** 2022-04-29

**Authors:** Chao Chen, Da-Hao Qu, Fang-Quan Shan, Ze-Xin Jin, Zhong-Shuai Sun

**Affiliations:** a Zhejiang Provincial Key Laboratory of Plant Evolutionary Ecology and Conservation, Taizhou University, Taizhou, China; b College of Life Sciences, Shanghai Normal University, Shanghai, China; c College of Life Sciences, Taizhou University, Taizhou, China; d Taizhou Green Heart Tourism Development Promotion Center, Taizhou, China

**Keywords:** *Lonicera crassifolia*, *Lonicera*, chloroplast genome, phylogenomics

## Abstract

*Lonicera crassifolia* is a prostrate or creeping, evergreen *Lonicera* species endemic to southwest China. Here, we reported the complete chloroplast (cp) genome of *L. crassifolia* (GenBank accession number: OK393707). The cp genome was 154,731 bp long, with a large single-copy region (LSC) of 88,619 bp and a small single-copy region (SSC) of 18,642 bp separated by a pair of inverted repeats (IRs) of 23,735 bp. It encodes 130 genes, including 85 protein-coding genes, 37 tRNA genes, and 8 ribosomal RNA genes. We also reconstructed the phylogeny of *Lonicera* using the maximum-likelihood (ML) method, including our data and previously reported cp genomes of related taxa. The phylogenetic analysis showed that *L. crassifolia* is a sister to the remaining Nintooa clade with strong bootstrap support.

The genus *Lonicera* L. is a major component of the family Caprifoliaceae, which comprises a large number of horticultural and economically important species (Ren et al. 2008; Jacobs et al. 2009). The genus includes 200 species of shrubs and climbers which are widely distributed in the Northern hemisphere (Rehder 1903, 1913; van Steenis 1946; Theis et al. 2008). *Lonicera* is well known for its taxonomic complexity, although has received extensive taxonomic evaluation and phylogenetic inference, the phylogenetic relationship among sections, subsections, and species is still unclear (Theis et al. 2008; Nakaji et al. 2015). *Lonicera crassifolia* Batalin 1892, which belongs to the section *Nintooa*, is a prostrate or creeping, evergreen shrub with axillary pink and yellow paired flowers, only occurs in streamsides, rocky cliffs, crevices of moist forest margins of southwest China (Hsu and Wang 1988). In order to better understand the phylogenetic relationship among the sections, subsections, and species of *Lonicera*, we assembled and characterized the complete chloroplast (cp) genome of *L. crassifolia* based on the Illumina pair-end sequencing data, and phylogenomic analysis of 20 *Lonicera* species was also presented.

Fresh leaves of *L. crassifolia* were collected from Wuguping, Xianfeng County, Hubei province (29.9860 N, 109.1126 E). A specimen was deposited at the Herbarium of Taizhou University (https://www.tzc.edu.cn/; collector: Zhong-Shuai Sun, sun2143998@163.com) under the voucher number LC1440-005. Total DNA was extracted from fresh leaves with a modified CTAB protocol (Doyle and Doyle 1987) and then sequenced using the Illumina NovaSeq platform (Illumina, San Diego, CA). The cp genome was assembled via NOVOPlasty (Dierckxsens et al. 2017), using the *Lonicera japonica* (NC026839) as the initial reference genome. The assembled cp genome was annotated using the online software GeSeq (Tillich et al. 2017) by comparing the sequences with the cp genome of *L. japonica*. Geneious R11 (Biomatters Ltd., Auckland, New Zealand) was used for inspecting the cp genome structure. The accurate annotated complete cp genome was submitted to GenBank with accession number OK393707.

The complete cp genome sequence of *L. crassifolia* is 154,731 bp in length, which has a characteristic quadripartite structure with a large single-copy (LSC) region of 88,619 bp, a small single-copy (SSC) region of 18,642 bp, and a pair of inverted repeats (IRs) of 23,735 bp. The overall GC contents of the total length, LSC, SSC, and IR regions were 38.6%, 37.0%, 33.4%, and 43.4%, respectively. The genome contained a total of 130 functional genes, including 85 protein-coding genes, 37 tRNA genes, and 8 rRNA genes.

Species phylogeny intra *Lonicera* and the phylogenetic position of *L. crassifolia* were evaluated based on protein-coding regions (CDS) of the cp genome. 84 shared CDS were extracted from a total of 26 complete cp genomes from Caprifoliaceae and aligned by MAFFT (Katoh et al. 2005). *Heptacodium miconioides* (NC042739) was used as an outgroup. We reconstructed a phylogeny employing the GTR + G model and 1000 bootstrap replicates under the maximum-likelihood (ML) inference in RAxML-HPC v.8.2.10 on the CIPRES cluster (Miller et al. 2010). A robust phylogeny of *Lonicera* was obtained based on the CDS data, and the genus was resolved as a monophyletic clade consisting of two well-supported clades, subgen. *Lonicera* and subgen. *Caprifolium* (Figure 1). Our result supports the classification of the two subgenera in *Lonicera* proposed by Rehder (1903, 1913) and Hsu and Wang (1988) and coincides with previous molecular phylogenetic studies (Theis et al. 2008; Nakaji et al. 2015; Wu et al. 2021). Among the four sections of subgen. *Lonicera* proposed by Rehder (1903, 1913) and Hsu and Wang (1988), sect. *Nintooa* was supported as monophyly, and *L. crassifolia* was placed at the base of the *Nintooa* clade with strong bootstrap support (Figure 1).

**Figure 1.**
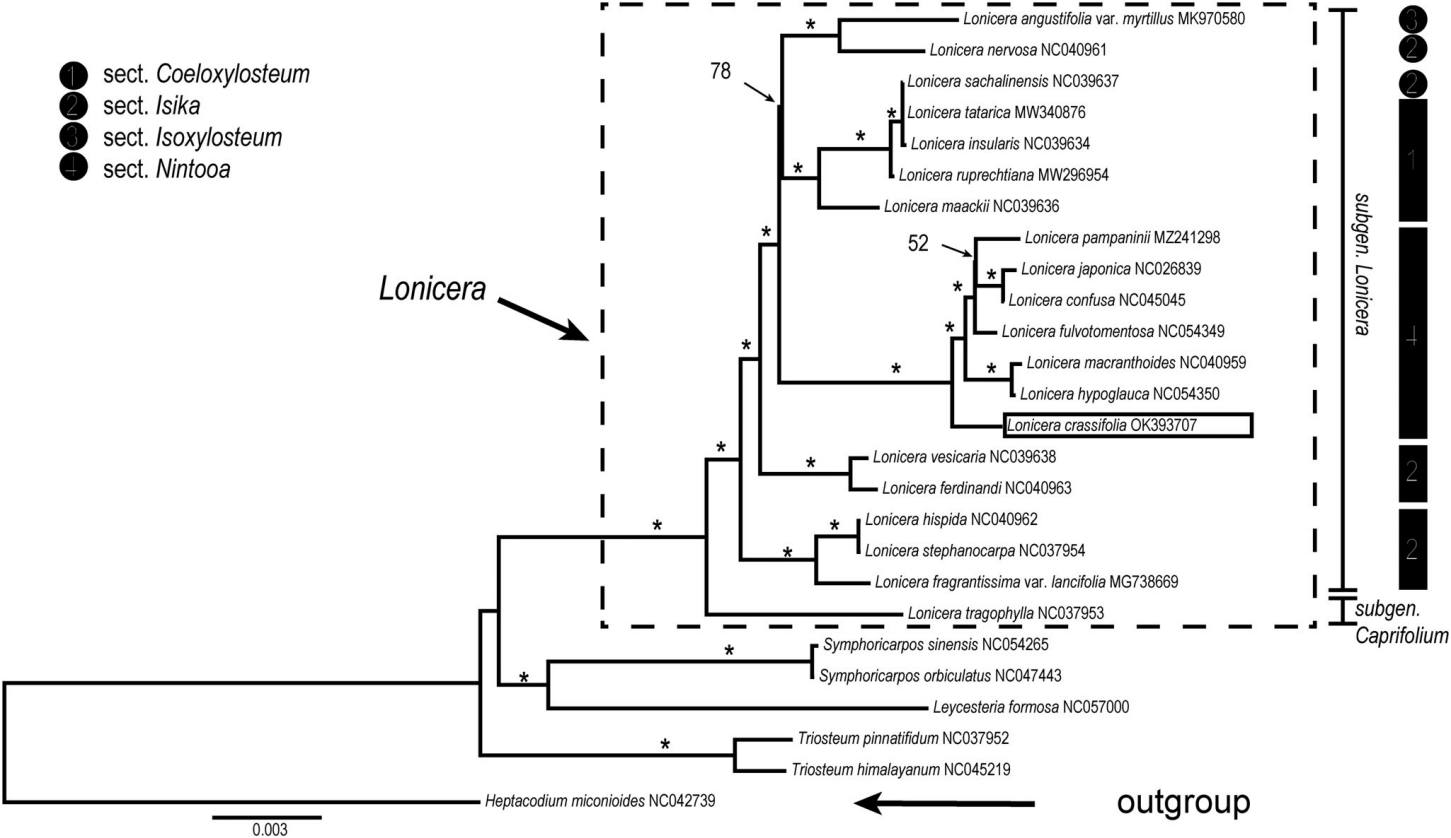
Maximum-likelihood (ML) tree reconstruction of 26 taxa from Caprifoliaceae based on 84 shared CDS in the chloroplast genomes. Relative branch lengths are indicated. Support values above the branches are ML bootstrap support; ‘*’ indicates 100% support values.

## Ethical approval

The material involved in the article does not involve ethical conflicts. This study was permitted by the Zhejiang Provincial Key Laboratory of Plant Evolutionary Ecology and Conservation, Taizhou University, China. All collection and sequencing works were strictly executed under local legislation and related laboratory regulations to protect wild resources.

## Authors’ contributions

The article was designed and conceived by Chao Chen and Zhong-Shuai Sun; Chao Chen and Da-Hao Qu assembled and annotated the cp genome; Fang-Quan Shan collected and identified the plant material; Zhong-Shuai Sun contributed significantly to phylogenetic analysis and manuscript preparation; Ze-Xin Jin was involved in the interpretation of the data and revised the manuscript critically for intellectual content. All authors approved the final version to be published and agreed to be accountable for all aspects of the work.

## Data Availability

The genome sequence data that support the findings of this study are openly available in GenBank of NCBI at (https://www.ncbi.nlm.nih.gov/) under the accession no. OK393707. The associated BioProject, SRA, and Bio-Sample numbers are PRJNA768984, SRR16235359 and SAMN22073044, respectively.
